# An unusual case of meningococcal meningitis complicated with subdural empyema in a 3 month old infant: a case report

**DOI:** 10.4076/1757-1626-2-6335

**Published:** 2009-09-17

**Authors:** Kawai Yip, Roly D Gosling, Victoria Jones, Ian K Hosein

**Affiliations:** 1 North Middlesex University Hospital Trust, Sterling Way, London, Greater London, N18 1QX, UK

## Abstract

Subdural empyema is an unusual complication of meningococcal meningitis, and in acute cases can be rapidly fatal. We present a case of an 8 week old infant who presented with atypical *Neisseria meningitis* with bifrontal subdural empyema formation. Through the utilisation of modern polymerise chain reaction tests on cerebrospinal fluid samples, we were able to confirm the diagnosis and institute appropriate treatment. Early surgical intervention and appropriate intravenous antibiotics meant that the patient fully recovered. In summary, early treatment of meningitis without adequate microbiological investigations can complicate later diagnosis of subdural empyema. Early suspicion of empyema should be considered when patient fails to improve after 48 hrs, seizures are a late sign and gives a poorer prognosis. Computed tomography scanning is still the modality of choice although in this case, magnetic resonance imaging had its benefits. Polymerase chain reaction of cerebrospinal fluid testing may also provide an important confirmatory test in future.

## Introduction

*Neisseria meningitidis* is a heterotrophic Gram-negative aerobic diplococcus with a polysaccharide capsule. Lipooligosaccharide is a component of the cell wall of *N. meningitidis* which acts as an endotoxin. Meningitis is a notifiable disease with which infection surveillance in the EU (EU-IBIS) has been recorded since 1999.

A literature search through Pubmed found only 9 cases of *Neisseria meningitis* with subdural empyema as a complication. The incidence of meningococcal disease is highest during the first year of life. Subdural empyema development in the course of meningococcal disease in adults is rare, but seems to affect children more frequently [1] Mortality ranges from less than 10% if patients are alert at the time of admission to 75% if the patient is already comatose.

Classical symptoms involve food refusal, fever, irritable, pale mottled skin and drowsiness [2]. The rest of the clinical manifestations were described according to the age of our patient, negative nuchal rigidity but bulging fontanelle. It may be for this reason that early symptoms of the subdural empyema were masked by meningitis. Other non-specific symptoms may have included altered mental state, papilloedema, seizures, cranial nerve palsies and hemiplegia. A rapid aggravation of focal symptoms is generally considered to differentiate it from slower progressing intracerebral abscesses.

## Case presentation

A previously well 8-week-old female identical twin baby, from an uncomplicated pregnancy, presented to the emergency department with a 2 day history of high fevers, irritability, diarrhoea, and decreased feeding which worsening over 24 hours. Mother and other family contacts, including identical twin, were asymptomatic.

On examination, the infant was pyrexial at 39°C, tachycardic with a capillary refill time of 4 seconds and a bulging anterior fontanelle. No obvious petechiae rash but skin mottling was seen. A clinical diagnosis of bacterial meningitis was made. Immediate management involved fluid resuscitation, septic screen and high dose intravenous ceftriaxone.

Laboratory studies showed a hemoglobin of 8.9 g/dl, a total white cell count of 2.9 × 10^9^/µl, with a neutropenia 0.9 × 10^9^/µl, C reactive protein (CRP) 266 and blood glucose 6.4 (units).

Lumbar puncture (LP) was performed after antibiotics; cerebrospinal fluid (CSF) was blood-stained and showed white cells of 30, red cells of 40,500 with 100% lymphocytosis. Gram stain was negative; CSF protein was 4.8 and glucose 0.5. Subsequently, Amoxicillin and acyclovir were added.

Despite 2 days of treatment the patient continued to spike fevers, although clinical symptoms improved. She developed a non-productive cough, with increased respiratory effort. Chest x-ray was normal. Both blood and CSF cultures were negative, and CRP had risen to 388. A repeated LP showed similar raised white cell count, Red cell count 4,500, 100% lymphocytosis, glucose 2.5, protein 4.5. and no organisms were seen on Gram stain.

A computed tomography (CT) of the head was performed revealing no obvious collection or abnormal pathology. Subsequent testing with abdominal ultrasound and trans-thoracic echocardiography (TTE) of her heart for a focal source for her sepsis revealed no abnormalities. However, CSF polymerase chain reaction (PCR) result from the reference laboratory showed a *Neisseria meningitidis*, confirming the diagnosis. The Health Protection Agency was notified and contact tracing took place.

On day 7, the patient began to suffer focal seizures, with jerking of her left arm and leg lasting 5 minutes. An urgent magnetic resonance imaging (MRI) scan without contrast of her head showed bifrontal and bitemporal subdural empyema with evidence of Leptomeningeal thickening. There was mild compression of the cortex without significant oedema, and as findings were bilateral there was no midline shift. Treatment was changed to intravenous Meropenem, with surgical intervention discussed. A craniotomy was performed and 3 ml of purulent pus was drained. PCR testing from this abscess was found to be *Neisseria meningitidis* serogroup B.

The infant responded well following surgery, seizures had stopped and treatment with antibiotics continued for a total course of 3 weeks. Sputum sample from the reference lab, following this showed Respiratory Syncytial Virus.

## Discussion

Subdural empyema is an unusual complication, occurring more commonly in meningitis caused by *S. pneumoniae* and *H. influenzae* type b rather than *N. meningitidis*. [3] Subdural empyema associated with *N. meningitidis* in infants was first reported in 1951 [4].

Routine testing (microscopy, culture, antigen detection) of meningococcal disease failed to identify any organisms in the majority of the 9 cases mentioned. This may have been due to the antibiotic treatment given prior to obtaining the CSF. The CSF changes were consistent with those of bacterial meningitis, i.e., turbid appearance, high cellularity, low glucose, high levels of proteins. Imaging studies provided an important adjunct to microbiology, and US/CT/MRI scanning have been advocated in many studies [5]. In terms of advancement, the modern utilisation of PCR testing for identifying bacterial DNA provides an effective tool in identifying specific organisms allowing for targeted therapy. Currently, the availability of Meningococcal PCR testing is uncommon throughout the UK, with question marks over its validity and costs. However, it is clear from this case that PCR provides a useful diagnostic tool in complicated cases. Rapid detection tests are being trialled in Australia (Quintain NS, 2007), based on nanoparticle and nanocluster technology.

The patient was treated initially with Ceftriaxone, as it was considered the drug of choice for empiric therapy according to the literature. Amoxicillin was added for *Listeria monocytogenes* cover, subsequent changes were based on clinical decisions. The three weeks of antimicrobial therapy in this case contrasts with the literature where duration of treatment is shorter, 4 or 7 days [6]. However, if we take into consideration the complications of these patients, 3 weeks of intravenous antibiotics were justified [5].

Epidemiologically, with the advent of Meningococcal C vaccine, introduced in 1999 and *H. influenza* vaccine, introduced in 2003, there has been a significant decline in meningitis. In countries with the vaccine, Meningococcal C incidence of 1.4 dropped to 0.1/100,000 in the period from 1999 to 2006. With this in mind, many authors now believe 5% of *N meningitidis* may be complicated with a subdural empyema [7,8].

Further investigation should be performed, as the aetiology of the infection is still unclear. Begging the question, why did one identical twin contract meningitis but other did not considering all environmental conditions were the same?

Another point to note was that this patient was non-vaccinated. The UK vaccination programme begins at 3, 4 and 12 months. The effectiveness of the vaccine is such that herd immunity to Men C has been identified in age groups, who have not been vaccinated, as bacterium carriage rates are reduced across the population [9]. Meningitis serotype B has been found to be responsible for 90% of lab confirmed cases, is associated with a higher mortality and currently comprises of 40% of UK cases.

## Conclusion

Young infants and children become ill very quickly. Identification of life threatening or organ threatening disease requires sequential observation. Meningitis is a dynamic disease, and early diagnosis offers a significantly improved prognosis. In cases where the patient has persistent pyrexia, despite adequate antibiotic treatment, it is vital to look for a focal source. Post-meningitis subdural empyema should be considered in all meningitic patients and should not be reserved only for *H. influenzae* or *S. pneumococcus*. In cases where CSF samples have failed to grow and contaminated CSF, Meningococcal PCR is recommended.

**Figure 1:**
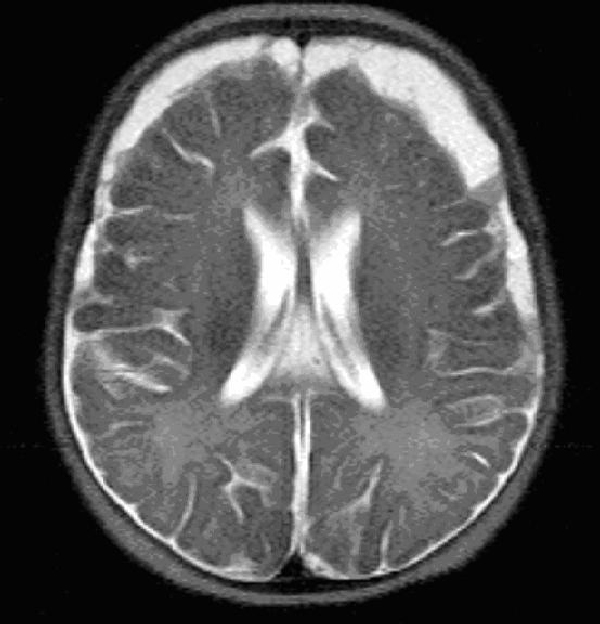
**MRI HEAD (axial) without contrast**. Meningitis related bifrontal/bitemporal subdural empyema. Notice the pronounced thickening up to 1.2 cm in the left side.

## Abbreviations

CRP: C- reactive protein; CSF: cerebrospinal fluid; CT: computed tomography; EU-IBIS: European Invasive Bacterial Infections Surveillance Network; MRI: magnetic resonance imaging; PCR: polymerase chain reaction; TTE: transthoracic echocardiogram.

## Consent

Written informed consent was obtained from the patient for publication of this case report and accompanying images. A copy of the written consent is available for review by the Editor-in-Chief of this journal.

## Competing interests

The authors declare that they have no competing interests.

## Author's contributions

KY witnessed case, wrote up case report and researched literature. RG, VJ and IKH acted as co-writers and analysts for results, providing insight into the aetiology of the disease. VJ cared for the patient.
